# Gaze Gesture Recognition by Graph Convolutional Networks

**DOI:** 10.3389/frobt.2021.709952

**Published:** 2021-08-05

**Authors:** Lei Shi, Cosmin Copot, Steve Vanlanduit

**Affiliations:** InViLab, Faculty of Applied Engineering, University of Antwerp, Antwerp, Belgium

**Keywords:** gaze, gesture recognition, graph neural network, graph convolution network, eye tracking

## Abstract

Gaze gestures are extensively used in the interactions with agents/computers/robots. Either remote eye tracking devices or head-mounted devices (HMDs) have the advantage of hands-free during the interaction. Previous studies have demonstrated the success of applying machine learning techniques for gaze gesture recognition. More recently, graph neural networks (GNNs) have shown great potential applications in several research areas such as image classification, action recognition, and text classification. However, GNNs are less applied in eye tracking researches. In this work, we propose a graph convolutional network (GCN)–based model for gaze gesture recognition. We train and evaluate the GCN model on the HideMyGaze! dataset. The results show that the accuracy, precision, and recall of the GCN model are 97.62%, 97.18%, and 98.46%, respectively, which are higher than the other compared conventional machine learning algorithms, the artificial neural network (ANN) and the convolutional neural network (CNN).

## 1 Introduction

Gaze gestures consist of sequences of eye movement elements (Drewes and Schmidt, 2007). Using gaze gestures is an effective hands-free modality for human–computer interaction (HCI) and human–robot interaction (HRI). The application of gaze gestures in HCI/HRI includes controlling a camera (Fujii et al., 2018), authentication (Findling et al., 2019), guiding a drone (Yu et al., 2014), text input (Wobbrock et al., 2008), and so on. Various approaches have been proposed for gaze gesture recognition. In Vaitukaitis and Bulling (2012) and Zhang et al. (2016), the gaze gestures are recognized based on the eye locations in the eye images. Gazture (Li et al., 2017) calculates the directions of gazes and extract the gaze gestures based on the directions of gazes.

Machine learning–based approaches are also used in recognizing the gaze gestures. In Fujii et al. (2018), the authors use gaze gestures to control a camera during laparoscopic surgeries. Kmeans and hidden Markov models (HMMs) are used to train and classify gaze gestures. One HMM model is trained for one gesture. The gaze points are first clustered by Kmeans clustering; the cluster features are then used to train the HMM model to recognize the gestures. In Rozado et al. (2012), the hierarchical temporal memory (HTM) algorithm is used to classify the gestures. The HTM captures the spatio-temporal relations of the gaze points and uses the Bayesian belief method to infer the gestures. In Findling et al. (2019), the authors evaluate the k-nearest neighbor (KNN), linear discriminant analysis (LDA), classification tree (CT), and support vector machine (SVM) on their gaze gesture data. Their results show that the SVM has the best performance on both opened eye gestures and closed eye gestures. In Chen et al. (2019a), the KNN, random forest (RF), extreme gradient boosting (XGBoost), SVM, artificial neural network (ANN), and convolutional neural network (CNN) models are evaluated and the CNN has the highest accuracy.

Deep learning has shown great successes in various fields (Dif and Elberrichi, 2020; Rath et al., 2021). Recently, graph neural networks (GNNs) (Scarselli et al., 2008) have drawn attention in the machine learning/deep learning communities. A graph consists of nodes and edges. The GNN propagates a node’s neighbors until convergence is reached. Due to the not permuted structure of a graph, that is, the number of the neighbors of different nodes may differ, convolutional operation cannot be applied in GNNs. One way to solve this problem is applying spectral approaches such as spectral networks (Bruna et al., 2013), CayleyNets (Levie et al., 2018), and graph convolutional networks (GCNs) (Kipf and Welling, 2016). Extensive reviews have been conducted regarding different types of GNNs; interested readers are referred to these reviews (Zhou et al., 2018; Wu et al., 2020).

GCNs are used in different applications including image classification (Chen et al., 2019), recommendation system (Ying et al., 2018), traffic forecasting (Yu et al., 2017), and text classification (Yao et al., 2019). In addition, GCN-based methods are also used for action recognition. In Yan et al. (2018), a spatial-temporal graph convolutional network (ST-GCN) is used to recognize skeleton-based actions. Graphs are constructed by human joints. The temporal dependencies are built by connecting the same nodes in the frame sequences. Several other studies (Si et al., 2018; Shi et al., 2019; Si et al., 2019) also use GNNs for skeleton-based action recognition. GCN is also used for action classification in videos (Wang and Gupta, 2018). Spatial graphs and temporal graphs are constructed and a GCN is used to classify an action in a video. The above-mentioned work shows that GCNs are proficient in classifying time series data. Gaze gesture is also in the form of time series data, which consists of sequences of data. One instance of gaze gestures could be transformed into a graph, and GCN-based model could be applied on graphs to classify the gaze gestures. Similar to deep learning, GCNs have the advantage of not requiring tailor-made preprocessing and feature selection compared to the conventional machine learning algorithms.

In this work, we propose a GCN-based model for gaze gesture recognition. The model consists of GCN layers, ResGCN (Li et al., 2019) layers and fully connected (FC) layers. The gaze gestures are converted to graphs and the graphs are trained by the GCN model for classification. We trained the proposed model on the HideMyGaze! dataset (Friström et al., 2019); the result showed that the GCN model has better accuracy than KNN, RF, SVM, ANN, and CNN.

GCNs have shown success in action recognition (Wang and Gupta, 2018; Yan et al., 2018); we want to introduce GCNs for the application in the eye tracking research field, specifically in gaze gesture recognition. To the best of the authors’ knowledge, this is the first study to apply a GNN for gaze gesture recognition. The main contribution of this work is using a model based on GCN for gaze gesture recognition. Previous studies have shown methods based on machine learning and deep learning (Fujii et al., 2018; Findling et al., 2019; Friström et al., 2019). We introduce a model based on state-of-the-art GNN, that is, GCN showing the potential for applying GCNs in eye tracking technology. The proposed model can also serve as a baseline model for evaluating other GNN models. The article is organized as follows. In Section 2, we explain how GCNs are used for gaze gesture recognition. In Sections 3 and 4, we show the details about the experiments and present the experimental results. We discuss results in Section 5, and the conclusion is drawn in Section 6.

## 2 Graph Convolutional Networks for Gaze Gesture Recognition

**FIGURE 1:**
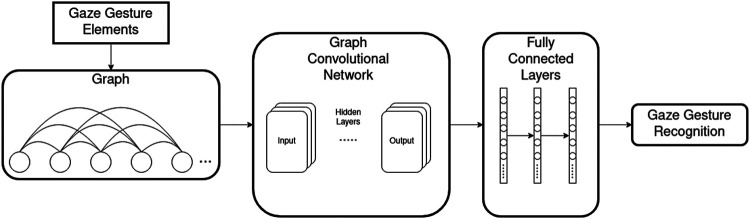
Overview of the GCN for gaze gesture recognition. The gaze gesture elements are converted into a graph. The nodes in the graph are densely connected (not all edges are displayed in this figure). The GCN takes the graph as input and FC layers are added for classifying the gaze gestures.

In this section, we describe how the gaze gestures are converted to graphs and use the GCN model to classify gestures. Figure 1 shows the gesture recognition system. The gaze gesture elements g in a gesture are formed into a graph first. Each gesture is represented by a graph. Then the graphs are passed to a GCN for extracting the graph features, FC layers are attached after the GCN for the final classification of the gaze gestures. A graph consists of nodes and edges. The mathematical representation of a graph G is as follows:

G=(V,ℰ,A), (1)

where V is the set of nodes and the ℰ is the set of edges in the graph. The adjacency matrix *A* describes the relations between the edges and the nodes. The nodes and the edges can be assigned with features.

A gaze gesture *g* consists of a set of gazes with features. For instance, if the gaze gesture is in the form of gaze points, the node feature will be the coordinate (x,y) of the gaze point. To convert a gaze gesture to a graph, each element in a gaze gesture is viewed as a node in the graph. The features of the elements are assigned as node features. Next, the edges are generated in the graph. For a node $v_{i}$ in the graph, it is connected to the next *k* nodes. Hence each gaze gesture is eventually transformed into a graph.

A GCN network f(G) takes the graph as inputs. The (i+1)th graph convolutional layer $H^{l+1},l\in \left(0,L\right)$ Kipf and Welling (2016) is as follows:

$$H^{\left(l+1\right)}=\sigma \left({\tilde{D}}^{-\frac{1}{2}}\tilde{A}{\tilde{D}}^{-\frac{1}{2}}H^{l}W^{l}\right),$$ (2)

where $\tilde{A}=A+I_{D}$ and $I_{D}$ is an identity matrix, $I_{D}$ represents the self-connected edges for every node in the graph. ${\tilde{D}}_{ii}={\text{Σ}}_{i}{\tilde{A}}_{ij}$ is a degree matrix. Both $\tilde{D}$ and $W^{l}$ are learnable. *σ* is the nonlinear activation function. A residual connection can also be added to the graph convolutional layer (Li et al., 2019), as follows:

$$H_{res}^{\left(l+1\right)}=\sigma \left({\tilde{D}}^{-\frac{1}{2}}\tilde{A}{\tilde{D}}^{-\frac{1}{2}}H^{l}W^{l}+H^{l}\right),$$ (3)

where $H_{res}^{\left(l+1\right)}$ is the output of a graph convolutional layer with residual connection. The GCN f(G) considers the neighboring nodes and gets a node feature representation. An ANN m(x) is stacked upon the GCN. To recognize the gesture, we perform a classification task, as follows:

Gesture=Softmax(m(f(G))), (4)

where Softmax function generates the classification scores and selects the class with the highest score as the recognized gesture.

## 3 Experiment

### 3.1 Dataset

We use the HideMyGaze! dataset (Friström et al., 2019) in the experiment. The dataset is used for authentication with gaze gestures. The dataset contains two sub-datasets, that is, the camera sub-dataset and the EOG sub-dataset. Both sub-datasets have closed eyes gestures and opened eyes gestures. For the closed eyes gestures, a gesture is performed when the human eyes are closed. The opened eye gestures are the gaze gestures performed when the eyes are opened. The camera sub-dataset is collected by Pupil-Labs eye tracking glasses and the EOG sub-dataset is collected by JINS MEME eye tracking glasses.

**FIGURE 2:**
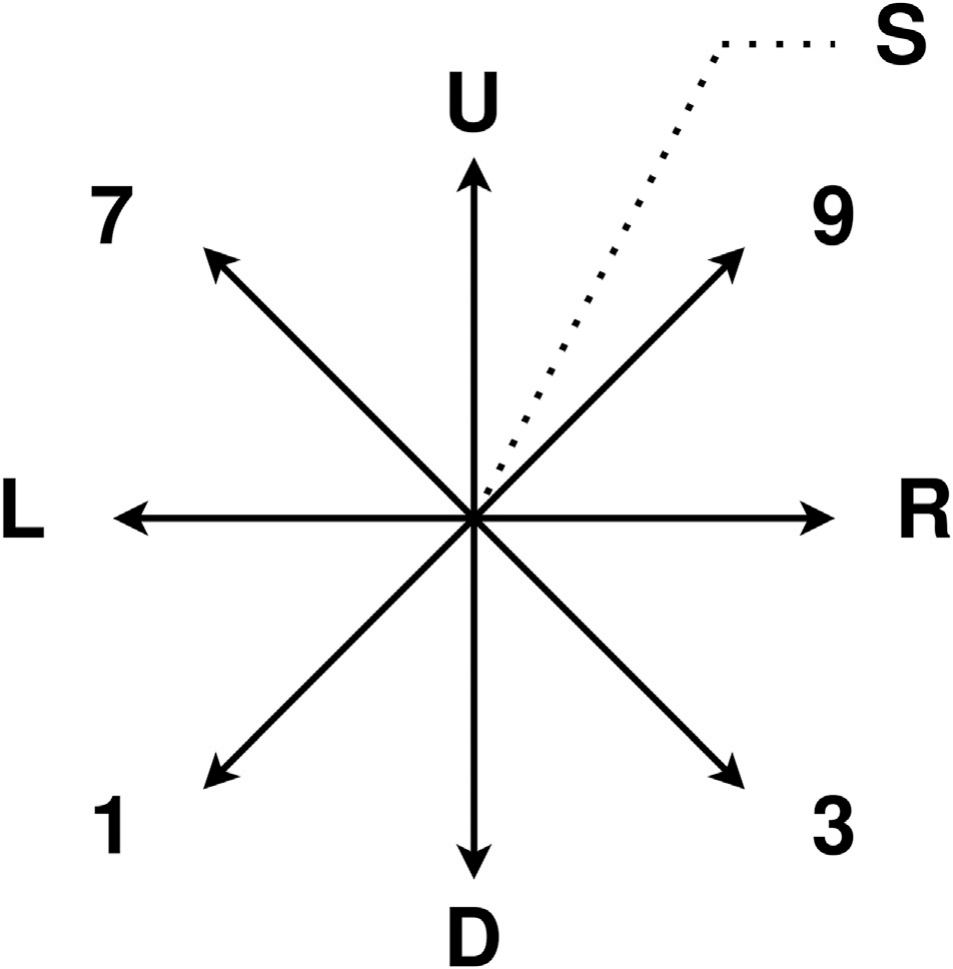
Gaze gestures in camera sub-dataset of the HideMyGaze! dataset. “U”: gesture in up direction. “D”: gesture in down direction. “L”: gesture in left direction. “R”: gesture in right direction. “1, 3, 7, 9”: gestures in diagonal directions. “S”: a squint movement.

We use the camera sub-dataset from HideMyGaze! dataset for the experiment. The dataset contains closed-eye gestures. The composition of gestures is shown in Figure 2. The gestures are in horizontal directions (“L”, “R”), vertical directions (“U”, “D”), diagonal directions (“1”, “3”, “7”, “9”), and a squint movement (“S”). The gesture is calculated from the eye image captured from the eye tracking glasses. The optical flows of the closed eye images are used as features. The dataset has 835 gestures in total, each sample has 12 features. The features are the mean optical flows in x and y directions and 10%, 25,% 50%, 75,% and 90%% quartiles of the optical flow in x and y directions.

### 3.2 Evaluation

We compare the proposed GCN model with several conventional machine learning algorithms and neural network models. To be specific, we compare our GCN model with KNN, RF, SVM, ANN, and CNN. We split the dataset into training set and test set with a ratio of 0.9. All models are evaluated by the classification accuracy, precision and recall, as follows:

$$Accuracy=\frac{total\;number\;of\;correct\;predictions}{total\;number\;of\;samples\;in\;the\;test\;set}.$$ (5)

$$Precision=\frac{\sum_{i=1}^{C_{l}}Precision_{i}}{C_{l}}.$$ (6)

$$Recall=\frac{\sum_{i=1}^{C_{l}}Recall_{i}}{C_{l}}.$$ (7)

Here, $C_{l}$ is the number of classes.

### 3.3 Implementation

The KNN, RF, and SVM are implemented with scikit-learn (Buitinck et al., 2013). The ANN and CNN are implemented with Pytorch. The GCN model is implemented with DGL library (Wang et al., 2019). Table.1 shows the architecture of the ANN, CNN, and GCN.

**TABLE 1:** Architectures of the ANN, CNN,and GCN models.

Model	Layers	Layer parameters
ANN	FC + LeakyReLu + Dropout	FC hidden units: 200
	FC + LeakyReLu + Dropout	Dropout rate: 0.5
	FC	
CNN	CNN + LeakyReLu + BN	CNN filters: 16, kernel size: 3, stride: 2
	FC + LeakyReLu + Dropout	FC hidden units: 200
	FC + LeakyReLu + Dropout	Dropout rate: 0.5
	FC	
GCN	GCN + ReLu + BN	GCN hidden units: 64
	ResGCN + ReLu + BN	ResGCN hidden units: 64
	FC + ReLu	FC hidden units: 64
	FC + ReLu	
	FC	

For the training of KNN, RF, and SVM, we perform grid searches for hyperparameter tuning. The distance and the number of neighbours of KNN are manhattan distance and three. For RF, the number of trees are 80 and the maximum depth is 80. For SVM, C=3 and γ=0.001.

To train ANN, the training epoch is 40 and the batch size is 32. The loss function is the cross entropy loss and the optimizer is Adam, and the weight decay of the optimizer is 0.004. The learning rate is $1e^{-4}$. CNN is trained with 120 epochs and batch size 32. The learning rate is $1e^{-4}$. We use cross entropy loss as loss function and Adam for optimization. For GCN, we train the model by 60 epochs. The batch size is 8. The loss function is the cross entropy loss. The optimizer is Adam and the weight decay is $4e^{-5}$. The learning rate is set to 0.001 and decay by a rate of 0.1 for every 30 epochs. The final model is the one with the best training accuracy.

## 4 Results

### 4.1 Effect of k

The GCN model takes graphs as inputs. As mentioned in Section 2, for a node $v_{i}$, connections are built between next *k* nodes in the graph. The effect of *k* for the GCN model is shown in Table 2. We test k=5 to k=25 with a step of 5 on the HideMyGaze! dataset. We perform a 5-fold cross validation on the training set and display the average accuracy. With larger *k*, there are more edges in the graph. When k=10, the average accuracy is the highest. With further increase of *k*, the average accuracy is not improving. We use k=10 for further evaluation.

**TABLE 2:** Effect of Different k in the graph of GCN and comparison with other algorithms on HideMyGaze! dataset.

*k*	Avg No. of edges	Avg accuracy
5	230	95.92%
10	495	96.45%
15	735	96.32%
20	950	96.05%
25	1,140	96.05%
	KNN	RF	SVM	ANN	CNN	GCN(*k* = 10)
Accuracy	83.33%	88.1%	91.67%	88.1%	94.05%	97.62%
Precision	75.62%	90.77	93.43%	89.41%	95.34%	97.18%
Recall	79.01%	85.03%	90.12%	87.5%	92.13%	98.46%

We show the average accuracy on 5-fold cross validation on the training set in the upper table and Accuracy on different algorithms in the lower table.

### 4.2 Comparison With Other Algorithms

Table 2 shows the evaluation results on the test set of the HideMyGaze! dataset. The proposed GCN model outperforms all other algorithms in accuracy, precision, and recall. The accuracy of GCN can achieve 97.62%, which is 3.57% higher than the one of the CNN model. The accuracy of SVM is also higher than 90%. The accuracies of the rest algorithms are all below 90%. The GCN model also has the highest precision and recall; they are 97.18 and 98.46%, respectively. CNN has the best precision (95.34%) and recall (92.13%). The precision and recall of SVM are also higher than 90%. Overall, the GCN model outperforms all other models in accuracy, precision, and recall. CNN has the second-best performance. RF and ANN have comparable results which is slightly lower than SVM. KNN has the lowest scores in all metrics.

## 5 Discussion

The GCN network takes graph as inputs. To convert a gesture to a graph, the elements in a gaze gesture are converted to nodes and the features of the elements are treated as the node features. It is not necessary to use hand-crafted features. Node features can be directly assigned by the element features. The edges between the nodes in graphs represent the temporal relations between the elements in a gaze gesture. For a node in the graph, we generate the edges for *k* next nodes. A larger *k* will generate more edges in the graph, however the accuracy is not linear to the *k*. If the gaze gestures are multi-modal sensory input (for instance gaze tracking together with electrooculography sensor), spatial-temporal graphs (Si et al., 2018) can be applied to adapt multi-modal data to a graph. The proposed model consists of a GCN network and FC layers, the GCN acts as the backbone of the model. The GCN can be interpreted as the learnable feature extractor on the graph level. Similar to deep learning, various GNN architectures (Zhou et al., 2018) can be used as the feature extractor or backbone. Although we did not test different GNN models, we have shown that GCN outperforms CNN and other conventional machine learning algorithms. Our proposed model can be used as a baseline model for gaze gesture recognition using GNN models.

## 6 Conclusion and Future Work

In this work, we propose a GNN-based model for gaze gesture recognition. The gaze gestures are converted to graphs and fed to a GCN-based model for classifying the gaze gestures. We train the model on the HideMyGaze! dataset and compare it with conventional machine learning algorithms as well as ANN and CNN. We have demonstrated the proposed model has the best performance in accuracy (97.62%), precision (97.18%), and recall (98.46%). We introduce a novel approach and provide a perspective, that is, GNN approach for gaze gesture recognition. Our experimental result show that it outperforms CNN, ANN, and several other machine learning approaches. We show that our approach has high potential for real gaze gesture applications. Our model also can be used as a baseline for evaluating different GNN architectures as feature extractors.

Using graphs to represent gaze gestures can establish custom temporal relations by adding edges between nodes. The GCN extract node features by considering neighbour nodes, it can deal gestures with different length without additional processing such as padding. One limitation is that the proposed model is not evaluated on large datasets. However, there is no large gaze gesture dataset available. We could collect our own dataset to evaluate the method, this is a part of the future work. Gaze gestures can be defined with different patterns and used for interaction with agents/computers/robots. One application is designing gaze gestures to move a robot in different directions. We will further develop this work by collecting our own dataset which has four gaze gestures. The four gestures are used to control a robotic manipulator in left, right, forward, and backward directions.

## Data Availability

Publicly available datasets were analyzed in this study. This data can be found here: https://ambientintelligence.aalto.fi/projects/hide_my_gaze/.
